# Evaluation of AFP surveillance indicators in polio-free Ghana, 2009–2013

**DOI:** 10.1186/1471-2458-14-687

**Published:** 2014-07-05

**Authors:** John Kofi Odoom, Nana Afia Asante Ntim, Badu Sarkodie, James Addo, Keren Minta-Asare, Evangeline Obodai, Miriam Eshun, Vincent V Ahove, Stanley Diamenu, Michael Adjabeng, Jacob Arthur-Quarm, Jacob S Barnor

**Affiliations:** 1 Department of Virology, Noguchi Memorial Institute for Medical Research, University of Ghana, P.O. Box LG 581, Legon, Accra, Ghana; 2 Disease Surveillance Department, Accra, Ghana; 3 World Health Organization, Country Office, Accra, Ghana

**Keywords:** Surveillance, Indicators, AFP, Regional Reference Polio Laboratory, Ghana

## Abstract

**Background:**

Ghana recorded the last case of indigenous wild poliovirus in 1999 but suffered two more outbreaks in 2003 and 2008. Following the World Health Organization (WHO) guidelines, transmission was interrupted through high routine immunisation coverage with live-attenuated oral polio vaccine (OPV), effective acute flaccid paralysis (AFP) surveillance and supplementary immunisation activities (SIA). This article describes the results of a five-year surveillance of AFP in polio-free Ghana, evaluate the surveillance indicators and identify areas that need improvement.

**Methods:**

We investigated 1345 cases of AFP from children aged less than 15 years reported to the Disease Surveillance Department from January 2009 to December 2013. Data on demographic characteristics, vaccination history, clinical presentation and virological investigation on stool specimens collected during investigation were analysed.

**Results:**

Of the specimens analysed, 56% were from males and 76.3% were from children less than 5 years of age. Twenty-four percent of the children received up to 3 doses of OPV, 57% received at least 4 doses while the status of 19% was unknown. Core AFP surveillance indicators were partly met for non-polio AFP rate while the WHO target for stool adequacy and timeliness was exceeded over the period of study. All the cases were classified virologically, however no wild polio was found. Sixty-day follow-up was conducted for 56.3% of cases and 8.6% cases classified as compactible with polio.

**Conclusion:**

Both laboratory and epidemiological surveillance for AFP were efficient and many WHO targets were met. However, due to the risk of poliovirus importation prior to global eradication, longterm surveillance is required to provide a high degree of confidence in prevention of poliovirus infection in Ghana. Thus, efforts should be made to strengthen regional performance and to follow–up on all AFP cases in order to establish proper diagnoses for the causes of the AFP leading to proper care.

## Background

The global effort to eradicate polio has become the largest public health initiative and is spearheaded by the World Health Organization (WHO) [1]. The characteristics of this infectious disease range in severity from a non-specific illness to severe flaccid paralysis with permanent disability [2]. It primarily infects children under 5 years of age and causes paralysis in one of every 200 to 1000 infections [3,4]. Infection is mostly by the faecal-oral route, typically in susceptible children without prior or adequate vaccination with potent and efficacious polio vaccines, living in areas of poor hygiene and sanitation. Nearly 95% poliovirus infection is asymptomatic with about 2% of people experiencing viral replication in the central nervous system which may lead to permanent neuronal damage and paralysis [5-7].

The WHO, in order to achieve eradication, recommended that countries conduct surveillance for cases of AFP which allows new cases to be identified where none had been before and can detect importations of wild polioviruses (WPVs). Since poliovirus is not the only agent that causes AFP, a broad case definition of all AFP including Guillian Barre syndrome, transverse myelitis, traumatic neuritis and transient paralysis associated with non-polio enteroviruses (NPEVs) infection among less than 15 years and all cases of suspected poliomyelitis among persons of any age is used. In order to maximise the ability of surveillance to rapidly detect imported cases, the established performance indicators for AFP surveillance requires that all cases of AFP be notified and investigated as prospective polio cases, including the collection of 2 stool samples 24 hours apart and within 14 days of the onset of paralysis [8,9]. Reporting should be complete, timely, and represent the geography and demography of the country and that follow-up examination for residual paralysis should be conducted in at least 80% of cases [10]. The surveillance system should also function to timely detect potential polio cases and alert health managers to institute appropriate interventions to interrupt poliovirus transmission and also validate the absence of wild poliovirus circulation in countries that are no longer reporting cases of polio [11,12].

The Expanded Programme on Immunization (EPI) was introduced in Ghana in 1978 [13] with childhood vaccination schedule for polio to include four doses of live-attenuated oral polio vaccine at birth, 6, 10 and 14 months of age. The Global Polio Eradication Initiative (GPEI) program was formally accepted in Ghana in 1996 after African Ministers of Health adopted the 1995 World Health Assembly (WHA) resolution urging member states to implement the GPEI. This strengthened the country to intensify routine polio immunization, implement supplementary immunization activities (SIAs), and introduce active AFP surveillance for poliovirus with full laboratory support [14,15].

The last known case of poliomyelitis caused by indigenous WPV transmission occurred in a 13-year old boy from Bole in the Northern region of the country on October 16, 1999 [16]. However, eight cases each due to importations from neighbouring countries caused polio outbreaks in 2003 and 2008 in Ghana. Following WHO guidelines for sensitive AFP surveillance and effective OPV immunisation, poliovirus transmission was interrupted and there has been no case(s) due to WPV. Subsequently, the country submitted documentation to the Africa Regional Commission on Certification (ARCC) in 2007 for recognition of polio free status after the break in transmission of WPV after the 2003 outbreak. Though the country is yet to be awarded a polio-free status, the thousands of AFP cases that continue to occur annually worldwide, with some associated with wild poliovirus [17] puts the country at risk of WPV re-importation. With the continuous collection of epidemiological data by the AFP surveillance system and regular annual meetings to update the surveillance officers, analysis of the data collected and evaluation of the programme is essential to identify gaps in the system to improve performance. This paper evaluates the AFP surveillance indicators and describes the results of a five-year surveillance for AFP cases in polio-free Ghana showing the progress made towards polio eradication from 2009 to 2013 as well as identifying aspects that require improvement.

## Methods

### Study design

Ghana has ten administrative regions with 216 districts. The health system is organized according to national, regional, district, sub-district, and community levels. The total projected population of the country for 2012, based on the growth rate of 2.5% from the 2010 national population census was 25,460,099 with children under 15 years representing about 42%.

A retrospective study was conducted using AFP surveillance data routinely collected between January 2009 and December 2013 by the Disease Surveillance Department of the Ghana Health Service and the WHO accredited Regional Reference Polio Laboratory (RRPL). All AFP cases reported to the Disease Surveillance Department of the Ghana Health Service during this period from all the ten regions were included in the study.

### AFP Surveillance

Ghana established AFP surveillance in 1996 to refine immunisation strategies to eradicate polio. When a patient meeting the AFP case definition is seen at a health facility, the health care practitioners conduct comprehensive investigations to rule out PV as a cause of the paralysis. These include taking a detailed history, conducting a systematic examination, and collecting two stool specimens, 24 to 48 hours apart, within 14 days of onset of symptoms. The case investigation form with information on demographic, clinical history, vaccination history and dates of stool specimen collection are completed to accompany the sample to the WHO-accredited RRPL at the Noguchi Memorial Institute for Medical Research for virus isolation and identification.

### WHO Accredited Regional Reference Polio Laboratory

The WHO-accredited RRPL in Accra, Ghana follows standardized protocols to; i) process stool samples to isolate PV, ii) identify PV to confirm WPV cases, iii) differentiate the three PV serotypes (1–3), WPVs, Sabin-like PVs and vaccine derived polioviruses (VDPVs) by intratypic differentiation (ITD) and iv) send the WPV and suspected VDPV isolates to a WHO Specialized Polio Laboratory to conduct genomic sequencing to monitor pathways of PV transmission by comparing the nucleotide sequence of the VP1 region of the genome from PV isolates [18]. The three standard laboratory timeliness indicators for stool specimen processing are to report ≥80% isolation results within 14 days of receipt, report >80% ITD results within 7 days of receipt of specimen and ship ≥80% of WPV and suspected VDPV isolates to the sequencing lab within 3 days of ITD results. The independent programmatic standard indicator is to report ITD results for ≥80% of isolates within 60 days of paralysis onset of persons with AFP cases; this indicator takes into account the entire interval from onset of paralysis through case notification, investigation, and specimen collection, transport, and testing. In addition to timeliness, the accuracy and quality of laboratory testing are monitored through an annual accreditation program of onsite reviews and proficiency testing [18].

### Final classification of AFP cases

**Figure 1:**
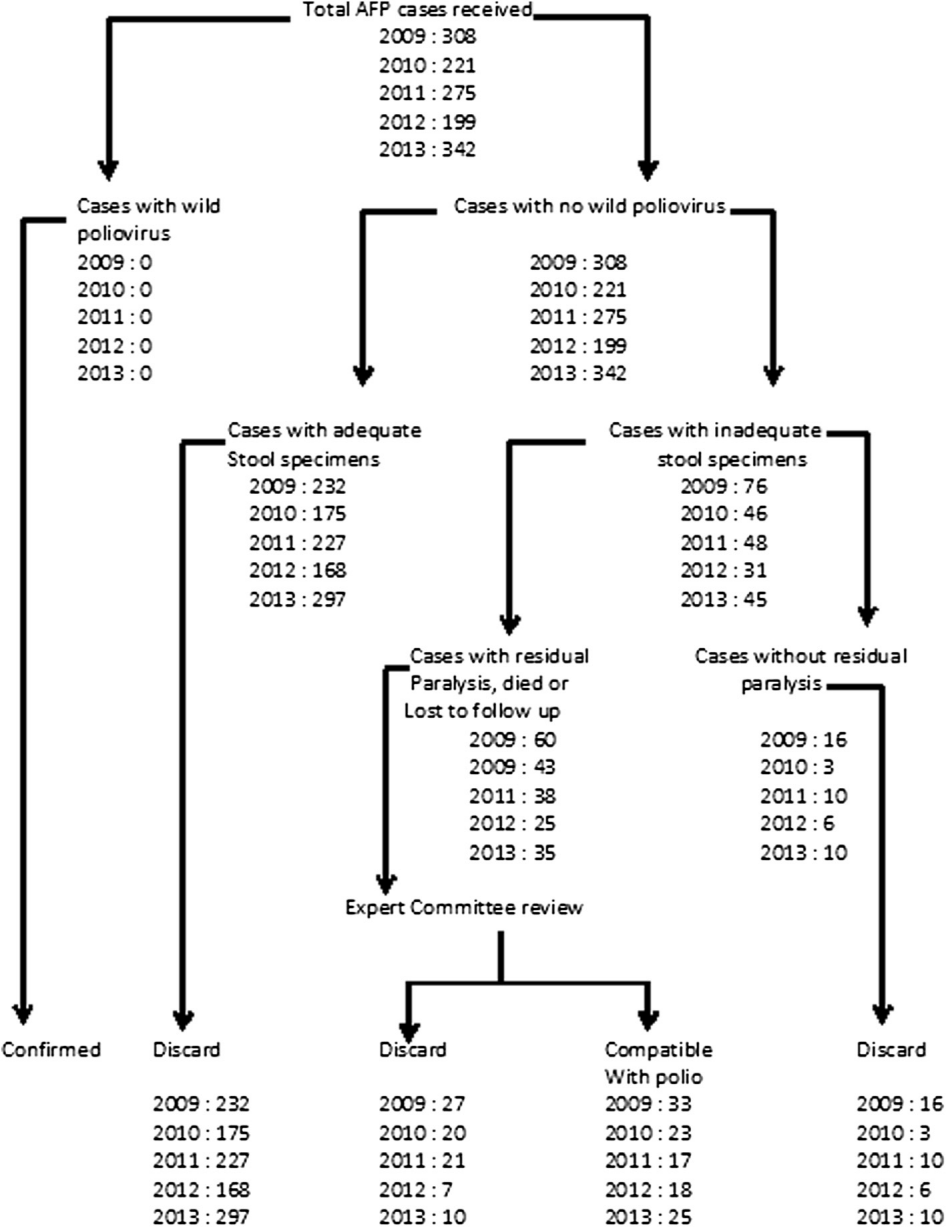
Virological flow chart of AFP cases classified in Ghana between 2009 and 2013.

The country has a National Polio Expert Committee (NPEC) that meets quarterly to conduct final classification of all AFP cases. An AFP case where two adequate stool specimens are submitted for analysis and no poliovirus is isolated is classified as a non-polio case (and is said to have been discarded). A case where the stool specimens are deemed inadequate but has no residual paralysis after 60 days of onset of symptoms is also classified as a non-polio case (discarded). A case that has inadequate stool specimens and has residual paralysis after 60 days of onset of paralysis or the patient is lost to follow up or dies within 60 days of symptom are classified as compatible with polio or discarded (Figure 1).

### AFP Surveillance indicators

The WHO has devised a set of performance indicators to ensure that AFP surveillance is properly maintained. We evaluated the quality of the AFP surveillance using the WHO guidelines for minimum performance standards [19-22].

### Data analysis

Demographic data was entered in an electronic database file (Microsoft Excel, 2003) and Epi Info version 3.5.1 (Centres for Disease Control and Prevention, Atlanta, United States). Frequencies and tables were generated using Microsoft Excel. To compare percentages, Pearson Chi- Square test were performed using SPSS 17.0. A p value <0.05 was considered as significant.

### Ethical considerations

Waiver for ethical approval for the study was obtained from the Institutional Review Board of the Noguchi Memorial Institute for Medical Research. Approval was also given by the Surveillance Department of the Ghana Health Service. We protected the confidentiality of patients through use of codes.

## Results

**Figure 2:**
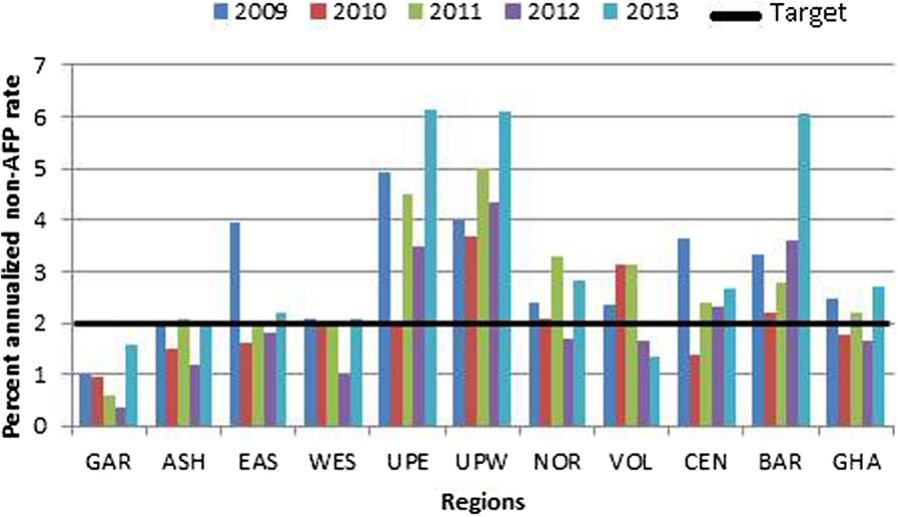
**Percentage of AFP cases with stool adequacy by year of the 10 region of Ghana, 2009–2013.** GAR-Greater Accra, ASH-Ashanti, EAS- Eastern, WES- Western, UPE- Upper East, UPW- Upper West, NOR- Northern, VOL- Volta, CEN- Central, BAR- Brong Ahafo and GHA- Ghana.

**Figure 3:**
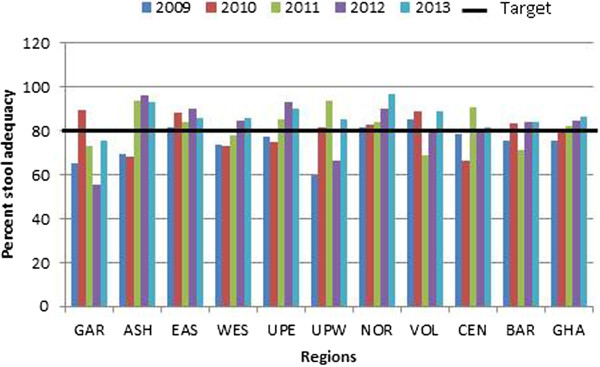
**Annualized non-polio AFP rate by year of the 10 region of Ghana, 2009–2013.** GAR-Greater Accra, ASH-Ashanti, EAS- Eastern, WES- Western, UPE- Upper East, UPW- Upper West, NOR- Northern, VOL- Volta, CEN- Central, BAR- Brong Ahafo and GHA- Ghana.

Stool samples from a total of 1, 345 AFP cases throughout the country collected from 2009 to 2013 were processed in the WHO accredited RRPL. All ten regions in the country provided samples within the five years of study with the majority from Ashanti Region. Of the specimens analysed, 56% were from males and 76.3% were from children less than 5 years of age (Table 1). The immunization status indicated that 24% of the children had received up to 3 doses of OPV, 54% had received at least 4 doses while the status of 19% of the children was unknown. As shown in Table 1, 1311 (97.5%) of the AFP cases had complete paralysis within 3 days of onset, 1133 (84.2%) had fever at the onset of paralysis and 871 (64.8%) had asymmetric paralysis.Figures 2 and 3 show the evolution of the more representative AFP performance indicators in Ghana during the period of study and include the performance target for each indicator established by WHO. The most important AFP indicator, non-polio AFP rate, which is the measure of the sensitivity of the system, reached acceptable values in 2009, 2011 and 2013. However, the rate of AFP cases due to causes other than poliomyelitis was well above the WHO target of 2 per 100,000 children under 15 years of age. Nevertheless, none of the ten regions consistently met the target for the period. Ashanti region reached the target in 2011 and 2013 while the Eastern and Western regions met the target in 2009. The Greater Accra region however, continuously failed to achieve the target throughout the period (Figure 2).The average national annual performance for the proportion of AFP cases with 2 adequate stool specimens fell below the 80% target in 2009 and 2010 but increased from 79.2% in 2010 to 82.5% in 2011 and to 86.7% in 2013 showing much improvement in the indicator. This was evident at the regional level where 5 of the 10 regions consistently met the target from 2011 to 2013. Of the 10 regions, six met the target in 2011, eight in 2012 and nine in 2013 indicating an improvement in the system. Nevertheless, the Greater Accra only met the target in 2010 while Western region achieve the target in 2012 and 2013 (Figure 3).

**Table 1:** Proportions of AFP by year, age, sex, OPV doses and clinical symptoms in Ghana, 2009-2013

**Variable**	**Year**				
**Age**	2009	2010	2011	2012	2013
<5	80.6	79.2	75.7	81.4	75.3
5-15	19.4	20.8	23.3	18.6	23.3
>15	0	0	1	0	1.4
**Sex**					
Male	55	53	56	59	56
Female	45	47	44	41	44
OPV doses					
≤3	25.6	27	21.9	23.5	20
≥4	58.4	53	58	58.5	60.5
Unknown	16.0	20	20.1	18	19.5
**Clinical condition**					
Sudden onset of paralysis	92.6	100	91	97	98
Fever	72.6	87.8	80	90.5	85.8
Asymmetrical	68.7	70	62	63.8	64.8

Despite the occasional slight drop in these two indicators, timeliness of arrival of samples to the WHO accredited polio lab was exceeded by the national for the study period (Table 2). The paradox of this comes to the fore when one considers the relative closer proximity of Greater Accra Region to the Polio laboratory as compared with other regions. Laboratory indicators including timeliness of reporting of virus isolation results, intratypic differentiation results, non-polio enterovirus isolation rate and sending of isolates for genomic sequencing were all met. The Polio laboratory was fully accredited over the period of study when it passed all the proficiency testing panels and on-site accreditation exercise except for 2009 when it was partially accredited for failing to obtain valid results for the cell sensitivity testing.

**Table 2:** AFP surveillance performance indicators in Ghanafrom 2008-2013

	**Year**					
**Parameter**	**Target**	**2009**	**2010**	**2011**	**2012**	**2013**
Number of AFP cases investigated	-	308	221	275	199	342
Annualized non-polio AFP rate 2/100,000 < 15 yrs population	≥2	2.48	1.83	2.2	1.64	2.69
Proportion of AFP cases with 2 stool specimens collected within 14 days of onset of paralysis	80%	75.4	79.2	82.5	84.4	86.7
Proportion of stool specimens arriving at national level within 3 days of being sent (%)	80%	86.6	87	84.7	85.9	86.0
Proportion of stool specimens arriving at national lab in good condition	90%	96.4	98.2	97.8	97.5	98.3
Proportion of stool specimens for which lab results were sent within 14 days of receipt at the lab (%)	80%	100	100	100	100	100
Proportion of stool specimens from which non-polio enterovirus was isolated (%)	10%	10	13.2	22.2	21.2	22

Final classification of all AFP cases using WHO virological classification by the Polio Expert Committee indicated that 36%, 27%, 16%, 28% and 25% of the districts did not report any AFP case in 2009, 2010, 2011, 2012 and 2013 respectively. Sixty-day follow up was conducted for 770 (57.2%) and significant (p = 0.008) proportion of the children were found with residual paralysis. Furthermore, the classification associated 21 (1.6%) and 17 (1.3%) cases of residual paralysis with Sabin-like poliovirus (P = 0.289) and non-polio enterovirus (P = 0.398) respectively with no statistical difference. No clear diagnosis was associated with the remaining 95 (7.1%) residual paralysis which could possibly be caused by Guillian-Barré Syndrome, Transverse myelitis or meningitis. Of the 1,345 AFP cases classified, 1105 (82.2%) cases from which adequate stools specimens were collected with no virus isolation from the lab and with no residual paralysis after 60-day follow up were discarded as non-polio AFP. The Committee further classified 116 (8.6%) of the cases as compactible with polio, 25 (1.9%) lost to follow-up and 14 (1%) died before follow-up was due (Table 3). None of the cases were classified as polio.

**Table 3:** Final classification of AFP cases by the National Polio Expert Committee

	**Final classification of AFP cases (%)**					**P-value**
	**2009**	**2010**	**2011**	**2012**	**2013**	
Expected AFP cases	102	204	216	222	223	0.001
Total received and classified	308 (302)	221 (108)	275 (127)	199 (89.6)	342 (153)	0.001
Discarded	275 (89.3)	175 (89.6)	227 (82.5)	168 (84.4)	297 (86.8)	<0.001
Cases followed up after 60 days	156 (50.5)	199 (58.2)	181 (53)	190 (55.8)	219 (64)	<0.001
Residual paralysis	60 (19.5)	43 (19.2)	38 (13.9)	25 (12.6)	55 (16.2)	0.008
Sabin	1	2	4	5	9	0.289
NPEV	3	4	1	1	8	0.398
Others	22	13	11	18	31	0.056
Compatibles	33	23	17	18	25	0.242
Lost to follow up	5	5	3	9	3	0.030
Died	2	5	3	2	2	0.072
Silent districts	61 (36)	46 (27)	27 (16)	48 (28)	43 (25)	0.001

P-value <0.05 is considered significant.

## Discussion

Our study represents a comprehensive national surveillance of AFP in Ghana and reports the results of a five-year evaluation of AFP surveillance indicators from 2009 to 2013 when the country did not report any wild poliovirus case(s).

We evaluated the age distribution of AFP cases which is also a risk factor and found those below the age of 5 years (59 months) to be 74.4%. This is consistent with a study conducted in Ibadan [23] which reported AFP cases in children <5 to be 74.3%, but higher than a report from Marches region (37%) in Italy [24] and lower than studies from Borno State [25] and India [26] which reported 82.5% and 90% respectively. The frequency of AFP was found to be consistently higher over the period of study among boys than girls with the mean incidence rate of 55.8% and 44.2% for boys and girls respectively but the difference was not statistically significant (p = 0.074). D’Errico *et al.* conducted a survey in Italy [24] and reported similar findings of higher incidence of AFP among boys than among girls. The study found higher proportion of the children with fever at onset of paralysis which progressed within 3 days with asymmetric paralysis, most occurring in the right-leg. We also evaluated symptom like fever at onset and found 97.5% of cases which was higher than that reported by Sevencan *et al.*[27], who found 15% of AFP cases with prodromal fever in Turkey.

Vaccination coverage with 4 doses of OPV among AFP cases was 80%. This was because parents and guardians of some children could not remember the number of OPV doses their children had taken or had misplaced the road-to-health cards though they claimed the children had received some OPV vaccination. This also indicates that the actual vaccine coverage with ≥4 doses could be higher than 80% perhaps giving no room for susceptible children who may help to sustain transmission of poliovirus in the country over the 5-year period. This is evident in Table 4 that OPV vaccination coverage for both routine and SIA are high. It is also significant to note that reduced immunization coverage may also have serious consequences in countries that use OPV, as was recently demonstrated by two outbreaks of wild poliovirus in Ghana in 2003 and 2008 when there was a reduced NIDs activity in 2002 and 2007 in the country [16].

**Table 4:** Summary of polio immunization activities in Ghana 2009-2013

**Year**	**NID/SNID**	**Date conducted**	**<5 yrs old targeted**	**<5 yrs reached**	**Reported coverage (%)**	**Routine immunization (%)**
2009	NID	12-14th Feb	4,759.19	4,781,445	100.5	88.7
2009	NID	27-29th Mar	4,759.19	4,988,591	102.1	
2010	NID	5-7 Mar	5,095,044	5,258,575	103.2	79.5
2010	NID	23-25 April	5,095,044	5,201,937	102	
2011	NID	24-26 Mar	5,285,575	5,462,530	103.9	86.7
2011	SNID	18-20 Aug	5,285,575	5,434,743	103.4	
2011	NID	27-29 Oct	746.089	757,993	102	
2012	NID	22-24 Mar	5,392,426	5,627,574	104.4	87
2013	NID	6-8 June	5,391,064	5,596,187	103.8	86
2013	NID	24-26 Oct	5,391,064	5,164,370	95.8	

*NID* National immunization days.

*SNID* Sub-national immunization days.

The results of the study further show that surveillance indicators have improved within the five-year study period. The over-all performance for stool adequacy rate increased from 2009 to 2010 and exceeded the WHO target from 2011 to 2013. However, the regional distribution of this quality indicator varied with only Eastern and Northern regions meeting the target throughout the five-year period while Greater Accra met it only in 2010. Additionally, analysis of our data indicated that the eight regions that could not meet their target throughout the five years failed to collect 2 separate specimens within 14 days of onset of paralysis. This is a surveillance failure and as Ghana pushes to be declared a polio-free country, regional surveillance officers must be encouraged to step-up their efforts to achieve the criteria once an AFP case was notified and investigated.

One of the criteria for polio-free certification is the detection and investigation of all cases of non-polio AFP in the population <15 years old. It is expected in such polio-free regions to have incidence of AFP to be at least 2 per 100,000 children aged <15 years old. The cumulative national performance only met the target in 2009, 2011 and 2013. Three regions; Brong Ahafo, Upper West and Upper East performed very well by meeting the target throughout the 5-year period but the overall national performance dropped because some regions under performed. The performance of six regions was inconsistent while Greater Accra region could not achieve the target in any of the years. It is possible that these regions can become pockets of transmission where polio virus circulation could go undetected if the virus is imported into the country. Efforts should therefore be made to strengthen the regional AFP surveillance system as well as sensitization of the medical staff towards the importance of detecting and reporting every AFP case to increase the sensitivity of the surveillance system.

WHO has specified timeliness of transportation of stool specimens for AFP surveillance, at least 80% of stool specimens to arrive at the polio laboratory within 72 hours of sending from the field [28]. Our results indicated that the country performed well above the target throughout the study period. This was made possible due to an efficient and special arrangements with the courier companies to collect specimens from the regional offices and directly send them to the polio lab. Another indicator used to evaluate the reliability and viability of stool specimen shipped to the laboratory for viral isolation is the non-polio enterovirus isolation rate. It is expected that at least 10% of stool specimens dispatched to the laboratory should yield NPEV. Our report shows that the national annual average of non-polio enterovirus isolation rate increased from 10% in 2009 to 13.2% in 2010 and had since remained above 20% from 2011–2013. Similar high NPEV isolation rate of 17.6% was reported in Egypt [29] 15.4% in Kerkuk [30], and 14.6% in Nigeria [31]. The polio laboratory has within the study period achieved full WHO accreditation apart from 2009 when it was partially accredited. Though the polio lab passed the proficiency test and the on-site visit exercise in 2009, it failed to obtain a valid result for the cell sensitivity testing and therefore was not fully accredited. Although the cell sensitivity is very crucial in the lab for the isolation of enteroviruses, valid test results are necessary to authenticate the test done and results obtained.

One of the limitations of this study was that we did not establish the exact diagnosis of AFP cases with negative stool specimens or the final diagnosis in a substantial majority of cases that left the children with residual paralysis. Final classification of all AFP cases by the National Polio Expert committee identified some silent districts that have population of 100,000 children less than 15 years not reporting any AFP case in each of the years. In such districts, poliovirus transmission and circulation could go undetected especially when herd immunity has not been achieved and there is an importation of wild poliovirus. Clinicians and surveillance officers in these areas should be trained and motivated to conduct active search for AFP cases. Using the virological classification set by WHO, no case(s) were classified as poliomyelitis but 116 cases were classified as compatible with polio. Clustering of compatibles occurred in Central region in 2010, Eastern region in 2011 and 2012 and in Greater Accra region in 2013. To reduce the number of compatibles in future, stool collection must be done within 14 days of onset of paralysis. Even though 14 Sabin-like polioviruses and 12 NPEVs left the children with residual paralysis, the cause for 76 residual paralyses was not determined. This is a setback to the program since AFP is a clinical condition with a wide range of possible differential diagnoses; accurate diagnosis of the cause of AFP is important for guiding therapy and prognosis. AFP case investigation should always aim at isolating the reason for the paralysis and the clinicians and surveillance officers should follow up all investigated cases and make certain that the final diagnoses are accurately captured on the AFP surveillance database [32].

## Conclusion

The study indicated that the AFP surveillance system was efficient over the past five years in Ghana, meeting most of the WHO established epidemiological and laboratory indicators. The system has its strength in timely transportation of stool specimen, stool specimens arriving in the lab in good condition, good laboratory performing standards, annual surveillance review meetings and up-to-date database. In addition to maintaining these best practices, the two most important AFP surveillance indicators, proportion of stool adequacy and non-polio AFP rates have to be strengthened to better enhance the overall performance especially in regions like Greater Accra which failed to meet most of the targets. Due to the risk of poliovirus importation prior to global eradication, longterm surveillance is required to provide a high degree of confidence in freedom from poliovirus infection in Ghana. The proportion of AFP cases follow up after 60 days of onset is deplorable and efforts should be made to follow all the AFP cases in order to establish proper diagnoses for the cause of the AFP leading to proper care. In this regard, clinicians, surveillance officers and health workers involved in the AFP surveillance activities have to be strengthened through sensitization meetings and motivation to detect, investigate, collect, transport, report and conduct 60-days follow up to fully meet the WHO standards for the eradication goals.

## Competing interests

The authors declare that they have no competing interests.

## Authors’ contributions

JKO participated in the study design, analysis of results and manuscript writing, NAAN was involved in sample processing and writing of manuscript, KMA and EO were involved in sample analysis and manuscript writing, BS participated in data interpretation and editing of the manuscript, JA participated in data analysis and editing of the manuscript, ME was involved in data analysis and manuscript writing, VVA was involved in the study design and interpretation of data, SD supervised the study protocol and interpretation of data, MA was involved in data analysis and interpretation, JAQ was involved in sample processing and editing of manuscript and interpretation and JSB participated in analysis and editing of data. All authors read and approved the final manuscript.

## Pre-publication history

The pre-publication history for this paper can be accessed here:

http://www.biomedcentral.com/1471-2458/14/687/prepub
